# The *in vitro* and *in vivo* anti-inflammatory and anti-oxidative effects of an amino acid blend supplemented feed on pigs experimentally challenged with *Salmonella Typhimurium*

**DOI:** 10.3389/fvets.2024.1367328

**Published:** 2024-03-13

**Authors:** Sehyeong Ham, Jeongmin Suh, Jieun Kim, Min Jeong Gu, Min Ah Park, Eunseon Oh, Jun-Ok Moon, Chanhee Chae

**Affiliations:** ^1^Department of Veterinary Pathology, College of Veterinary Medicine, Seoul National University, Seoul, Republic of Korea; ^2^Application Center, CJ Blossom Park, Suwon, Republic of Korea

**Keywords:** amino acid blend, cytokine, inflammation, *Salmonella Typhimurium*, porcine

## Abstract

**Background:**

The *in vitro* and *in vivo* anti-inflammatory and anti-oxidative effects of an amino acid (AA) blend (tryptophan, threonine, and methionine) in pigs.

**Objective:**

This study aimed to evaluate the *in vitro* anti-inflammatory and anti-oxidative effects of an AA blend on intestinal porcine epithelial cells (IPEC-J2) and the *in vivo* anti-inflammatory and anti-oxidative effects in pigs experimentally challenged with *Salmonella Typhimurium*.

**Methods:**

IPEC-J2 were pretreated with an AA blend for 25 h and then treated with lipopolysaccharide (LPS), deoxynivalenol (DON), or H_2_O_2_ for *in vitro* evaluation. A controlled standard diet supplemented with 0.3% of the AA blend was orally fed to the treated group pigs for 14 days, beginning at 21 days of age. At the end of the feeding period, pigs were orally inoculated with *Salmonella Typhimurium*.

**Results:**

Pre-treatment with the AA blend reduced LPS/DON-induced interleukin (IL)-8 mRNA as a measurement of the anti-inflammatory effect and H_2_O_2_-induced reactive oxygen species (ROS) as a measurement of the anti-oxidative effect on IPEC-J2. Feeding with an AA blend resulted in a reduction of proinflammatory (tumor necrosis factor-α, IL-6, and IL-8) cytokine levels, while treated pigs experienced an increase in anti-inflammatory IL-10 cytokine in their sera. The addition of an AA blend-supplemented pig feed resulted in significantly lower *Salmonella*-induced cecal lesion scores compared to untreated pigs.

**Discussion:**

Supplementation of feed with an AA blend reduced intestinal inflammation and pathology in pigs and may be applied for the control of *Salmonella Typhimurium* infection, as demonstrated in this study.

## Introduction

The most widely spread of all salmonellae is *Salmonella enterica* serovar Typhimurium (*S. typhimurium*), which is associated with enterocolitis and is the second most frequently isolated serotype from pigs (1). Necrosis of cryptic and surface enterocytes (whether local or diffuse) is revealed during histopathological examination for signs of this infection. During the acute stages of the disease, the lamina propria and submucosa contain neutrophils. *Salmonella Typhimurium* promotes its own colonization through the exploitation of inflammation (2). This *S. typhimurium*-induced intestinal inflammation overcomes colonization resistance with a profound dysbiosis of the colonic microbial community structure (3, 4).

Pigs experimentally infected with *Salmonella Typhimurium* displayed an acute inflammatory response (5). *Salmonella* infection induces the expression of various inflammatory cytokines (6). TNF-α is expressed during *Salmonella* infection and is associated with the host’s inflammatory responses in the intestinal tract (6). Systemic and mucosal antibody response development occurs through the production of interleukin (IL)-6 by TNF-α production (7). *Salmonella Typhimurium* produces IL-8, the main function of which is to act as a neutrophil chemo-attractant and activating factor while stimulating the production of proinflammatory cytokines (8, 9). On the other hand, the expression of IL-10 is also increased in the intestinal tract during *Salmonella* infection, where it downregulates inflammatory responses (6). In addition to inflammatory cytokines, reactive oxygen species (ROS) participate in the progression of an inflammatory reaction. An enhanced ROS generated by neutrophils at the site of inflammation is involved in damage to vascular endothelial cells and tissue injury (10).

Controlling inflammation may therefore be the key to preventing *S. typhimurium* infection in pigs. Amino acids (AAs) and their use in the intestinal tract are gaining attention as inflammatory regulators. Three of the key essential AAs for pigs are methionine (Met), threonine (Thr), and tryptophan (Trp) (11–13), and each plays an important role in inflammation regulation in growing pigs (12, 14). Although supplementation of piglet diets with tryptophan reduced the negative impact of *Escherichia coli* K88 at weaning, supplementation with threonine was not sufficient to reduce the negative impact of *S. typhimurium* (15, 16). The present study evaluated the anti-inflammatory effect of three AAs combined into a feed additive blend (Trp + Thr + Met) provided in pigs experimentally challenged with *S. typhimurium*.

## Materials and methods

### Ethical statement

All of the methods were previously approved by the Seoul National University Institutional Animal Care and Use Committee (Approval No. SNU-220621-3).

### Preparation of an amino acid blend

The AA evaluated in this study were blended to contain L-methionine (L-MET eco, 95% purity, CJ Cheiljedang Co., Ltd.), L-threonine (THR Pro, 80% purity, CJ Cheiljedang Co., Ltd.), and L-tryptophan (TRP Pro, 60% purity, CJ Cheiljedang Co., Ltd.). Tryptophan and threonine were granule-type feed-grade AA that were produced in *Corynebacterium glutamicum*. The AA blend was created at a weight ratio of 55 (Met):43 (Thr):2 (Trp).

### Cell culture

Intestinal porcine epithelial cells (IPEC-J2, DSMZ No. ACC701, BWE, Germany) were seeded in T75 cell culture flasks (70,075, SPL Lifesciences, Gyeonggi-do, Korea) and cultured with Dulbecco’s Modified Eagle Medium/Nutrient Mixture F-12 (DMEM/F-12, Thermo Fisher Scientific, Waltham, United States) containing 10% fetal bovine serum (FBS, SV30207.02, HyClone, Cytiva, Australia), 1% insulin–transferrin–selenium–ethanolamine (ITS-X, 51500-056, Thermo Fisher Scientific, Waltham, United States), and 1% penicillin–streptomycin (15140-122, Thermo Fisher Scientific, Waltham, United States) at 37°C in a humidified atmosphere with 5% CO_2_. The cells were sub-cultured once every 3 to 4 days (twice a week) until the confluence reached 90%. For sub-culture, the cells were detached from the flask by trypsin/EDTA (GIBCO, NY, United States), centrifuged at 300 × *g* for 3 min, re-suspended, and re-seeded at a concentration of 5.0 × 10^5^ cells in 25 mL of complete cell culture medium per T75 flask (17).

### *In vitro* evaluation of the anti-inflammatory effect of an AA blend

IPEC-J2s were seeded at 1.0 × 10^5^ cells/well (1 mL per well) in 24-well plates and cultured overnight. Cells were pretreated with or without AA blend for 24 h and challenged with 1 μg/mL of deoxynivalenol (DON, D0156, Sigma Aldrich, St. Louis, United States) and 1 μg/mL of lipopolysaccharide (LPS, L2630, Sigma Aldrich) for 3 h.

After these treatments, the cells were washed with cold PBS and harvested for total RNA extraction and the quantification of IL-8 mRNA. The percent reduction of IL-8 was calculated following the equation below:


$$\mathrm{Reduction}\;\left(\%\right)=\;\left(\frac{\begin{array}{l} RGE,\mathrm{challenged}\;\mathrm{cells}-RGE,\\ \mathrm{pretreated}\;\mathrm{and}\;\mathrm{challenged}\;\mathrm{cells} \end{array}}{RGE,\mathrm{challenged}\;\mathrm{cells}}\right)\times 100$$

where RGE is relative gene expression.

### RNA extraction and real-time PCR for IL-8 mRNA expression

Total RNA was extracted from IPEC-J2 using the easy-spin RNA extraction kit (17,221, iNtRON, Korea). The RNA purity and concentration were measured using spectrophotometry (QIAxpert System, Qiagen, Hilden, Germany).

A quantitative reverse transcription PCR (RT-qPCR) was performed on the Rotor-Gene Q 2plex platform (Qiagen) in a final volume of 20 μL using the AccuPower GreenStar RT-qPCR PreMix (K-6403, Bioneer, Korea). The PCR reaction mixture contained 10 μL of 2X master mix, 2 μL of each primer (10 pmol/μL), 2 μL of template RNA, and 4 μL of DEPC-DW. The mixture was added into a PCR tube, and the PCR consisted of cDNA synthesis at 50° C for 15 min and pre-denaturation at 95° C for 5 min, followed by 40 cycles at 95\u00B0C for 15 s, 55° C for 30 s, and 72° C for 30 s. The primers were designed with Primer-Blast^1^ based on the published cDNA sequence in the Gene Bank. The relative abundance of targeted genes was calculated according to the 2^−ΔΔCt^ method and normalized to the mean expression of GAPDH, which serves as the internal reference gene. Information on the detected genes and primers is shown in Table 1.

**Table 1:** Primer sequences used in this study.

Gene	Primer sequences (5′-3′)	Length, bp	Access no.
IL-8^1^	F: AGCCCGTGTCAACATGACTT R: TGGAAAGGTGTGGAATGCGT	147	NM_213867.1
GAPDH^1^	F: GTTGTGGAGTCCACTGGTGT R: CCCATCACAAACATGGGGGC	119	NM_001206359.1

1 IL-8, interleukin-8; GAPDH, glyceraldehyde 3-phosphate dehydrogenase.

### *In vitro* evaluation on the antioxidant effect of an AA blend

IPEC-J2s were seeded at 1.5 × 10^4^ cells/well (100 μL per well) in a 96-well plate. After overnight incubation at 37°C, cells were pretreated with or without the AA blend for 18 h and then washed with Hank’s Balanced Salt Solution (HBSS, 14175095, Thermo Fisher Scientific, Waltham, United States). To determine the amount of ROS, cells were incubated with 10 μM DCF-DA (2′, 7′-dichlorofluorescin-diacetate, D6883, Sigma Aldrich, St. Louis, United States) for 30 min and then treated with 0.5 mM H_2_O_2_ for 30 min. The fluorescence was read at 485 nm for excitation and 530 nm for emission with a fluorescence microplate reader (BioTek Synergy H1). The percent reduction of ROS was calculated following the equation below:


$$\mathrm{Reduction}\;\left(\%\right)=\;\left(\frac{\begin{array}{l} RFU,\mathrm{challenged}\;\mathrm{cells}-RFU,\\ \mathrm{pretreated}\;\mathrm{and}\;\mathrm{challenged}\;\mathrm{cells} \end{array}}{RFU,\mathrm{challenged}\;\mathrm{cells}}\right)\times 100$$

where RFU is the relative fluorescence unit.

### Salmonella strain

The strain of *S. enterica* used in this study belongs to serovar Typhimurium, derived from the pig-virulent strain KVCC-BA1300432. This strain was kindly supplied by the Korean Veterinary Culture Collection (Gimcheon-si, Gyeongsangbuk-do, Republic of Korea).

### *In vivo* experimental design in pigs

Twenty-four 21-day-old pigs were purchased from a Salmonella-free herd. A commercial ELISA test (Swine *Salmonella* Ab Test, IDEXX Laboratories Inc., Westbrook, ME, United States) was used to test pig serology for *Salmonella* upon arrival at the Seoul National University facility. Twenty-four, 21-day-old pigs were randomly distributed into three groups (8 pigs per group). The diet was formulated according to the nutritional recommendations (18, 19).

At −14 days post-challenge (dpc, 21 days of age), pigs in the AA+ST group were started on a controlled standard diet feed supplemented with 0.3% of an AA blend. At 0 days post-challenge (dpc, 35 days of age), pigs in the AA+ST and ST groups were orally inoculated twice within 4 h using 1 mL of a growth medium containing 3.3 × 10^9^ CFU/mL of *S. enterica* serovar Typhimurium. Pigs in the control group were orally inoculated twice within 4 h with 1 mL of sterile saline solution. At 14 dpc (49 days of age), pigs were sedated by an intravenous injection of sodium pentobarbital and then euthanized by electrocution as previously described (20). Tissues were collected from each pig at necropsy.

### Sampling collection

Blood samples were collected from all pigs at −14, 0, 3, 7, and 14 dpc.

### Clinical observations

Rectal temperatures were recorded at 0, 1, 2, 3, 4, 5, 6, 7, and 14 dpc at the same time by the same personnel. A fecal scoring system was defined according to the following scale: 0 (normal), 1 (semisolid feces without blood), 2 (watery feces without blood), and 3 (blood-tinged feces) (4).

### Body weight and average daily weight gain

Pig weight was measured at −14 (21 days of age), 0 (35 days of age), and 14 (49 days of age) dpc throughout the study. An average daily weight gain (ADWG = grams/pig/day) was calculated over three time points: (i) between −14 and 0 dpc, (ii) between 0 and 14 dpc, and (iii) between −14 and 14 dpc at the study conclusion. The difference between the initial final weights was divided at each of these three time points by the number of days in the corresponding period to calculate ADWG. All data were obtained in a blinded manner.

### Cytokine assay

Serum samples were collected for the quantification of tumor necrosis factor-α (TNF-α), IL-6, IL-8, and IL-10, as assayed using a commercial ELISA kit (Porcine TNF-alpha Quantikine ELISA Kit, Porcine IL-6 Quantikine ELISA Kit, Porcine IL-8/CXCL8 Quantikine ELISA Kit, and Porcine IL-10 Quantikine ELISA Kit, R&D Systems, Inc., Minneapolis, MN, United States). The results were expressed as pg./mL.

### Superoxide dismutase activity

Serum superoxide dismutase (SOD) activity was measured using commercial kits (OxiSelect™ Superoxide Dismutase Activity Assay Kit, Cell Biolabs, Inc., San Diego, CA, United States).

### Protein carbonyl and malondialdehyde assay

A commercial protein carbonyl fluorometric assay was used for the measurement of protein carbonyl content in serum (Cell Biolabs, Inc., San Diego, CA, United States). A commercial thiobarbituric acid reactive substances assay kit was used for the direct quantitative measurement of malondialdehyde in serum samples.

### Salmonella lesion score

A scoring system for microscopic cecal lesions was defined according to the following scale: 0 (normal), 1 (mild neutrophilic infiltrate without submucosal infiltrates), 2 (moderate neutrophilic infiltrate with or without submucosal infiltrate), and 3 (marked neutrophilic infiltrate with or without submucosal infiltrate) (21).

### Statistical analysis

For the *in vitro* experiment, differences among the experimental data were assessed using a one-way ANOVA, followed by Tukey’s *post-hoc* test and F-protected test. For the *in vivo* experiment, a normal distribution was determined with the Shapiro–Wilk on these data. Whether or not the groups had statistically significant differences between them at various time points was then determined by performing a one-way ANOVA. For further evaluation, a *post-hoc* test for a pairwise comparison with Tukey’s adjustment was conducted with a statistical significance result from the one-way ANOVA test. A Kruskal–Wallis test was additionally performed only in cases where the normality assumption was not met. The results that showed statistical significance from the Kruskal–Wallis test were further evaluated with the Mann–Whitney test to compare the differences among the groups. The results were reported in *p*-values, and p-values of <0.05 were considered significant.

## Results

### *In vitro* anti-inflammatory effect of an AA blend

**Figure 1:**
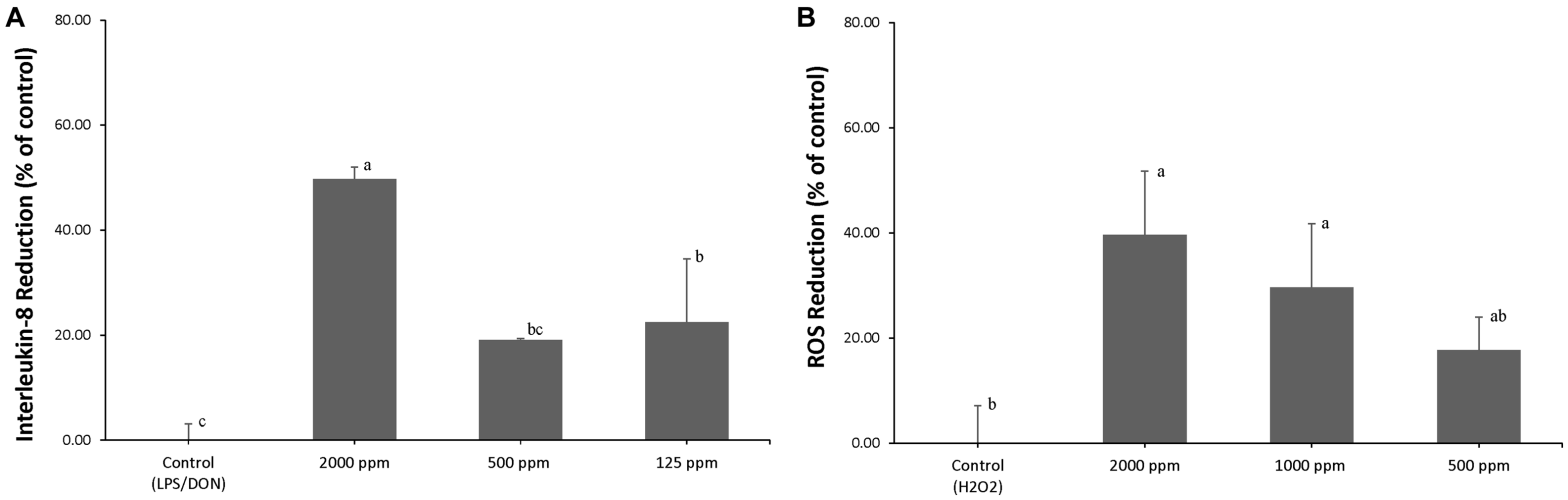
**(A)** IPEC-J2 were pretreated with an AA blend (0, 125, 500, and 2,000 ppm) for 24 h, and inflammation was induced by exposure to 1 μg/mL LPS and 1 μg/mL DON. Percent (%) of IL-8 reduction was calculated by (RGE of challenged cells – RGE of pretreated and challenged cells)/RGE of challenged cells × 100, where RGE is relative gene expression. Different letters mean statistically significant differences (*p* < 0.05). **(B)** IPEC-J2s were pretreated with an AA blend (0, 500, 1,000, and 2,000 ppm) for 24 h, and oxidative stress was induced by exposure to 0.5 mM H_2_O_2_. Percent (%) of ROS reduction was calculated by (RFU of challenged cells – RFU of pretreated and challenged cells)/RFU of challenged cells × 100, where RFU is relative fluorescence units (*p* < 0.05).

AA blend treatment significantly reduced (*p* < 0.05) LPS/DON-induced IL-8 expression to 49.7% ± 2.3% (2,000 ppm of AA blend), 19.1% ± 0.3% (500 ppm of AA blend), and 22.5% ± 12.1% (125 ppm of AA blend) compared to that of control (Figure 1A).

### *In vitro* antioxidant effect of an AA blend

AA blend treatment significantly reduced (*p* < 0.05) H_2_O_2_-induced ROS to 39.7% ± 12.1% (2,000 ppm of AA blend), 29.6% ± 12.1% (1,000 ppm of AA blend), and 17.7% ± 6.3% (500 ppm of AA blend) compared to that of control (Figure 1B).

### Clinical observations

**Figure 2:**
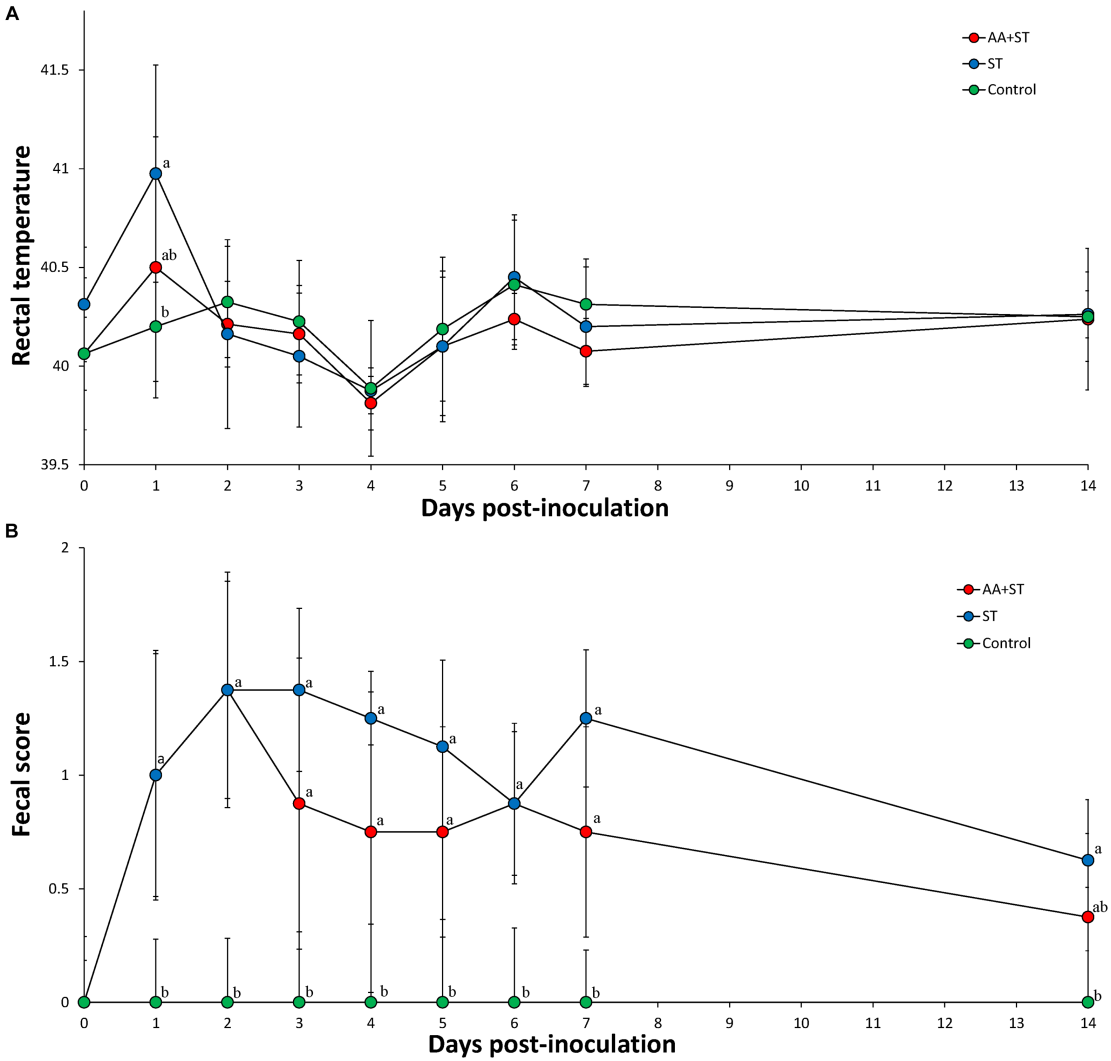
**(A)** Body temperature. **(B)** Fecal score. Variation is expressed as the standard deviation. Pigs were fed a control standard diet supplemented with an amino acid blend, followed by oral inoculation with *Salmonella enterica* serovar Typhimurium (AA+ST group), oral inoculation with *Salmonella enterica* serovar Typhimurium without supplementation with an amino acid blend (ST group), and control pigs. Different letters within a sampling point mean statistically significant differences (*p* < 0.05).

The mean rectal temperature was significantly higher (*p* < 0.05) in pigs (40.98 ± 0.66) from the ST group at 1 dpc compared to those of the control group pigs (40.20 ± 0.28) (Figure 2A). Pigs in the AA+ST and control groups had significantly (*p* < 0.05) lower fecal scores than those in the ST group at 1 to 7 dpc. Pigs in the control group had significantly (*p* < 0.05) lower fecal scores than those in the ST group at 14 dpc (Figure 2B).

### Growth performance

The body weight and ADWG of the pigs were measured at −14, 0, and 14 dpc, where significant differences among the three groups were not found.

### Cytokine assay

**Figure 3:**
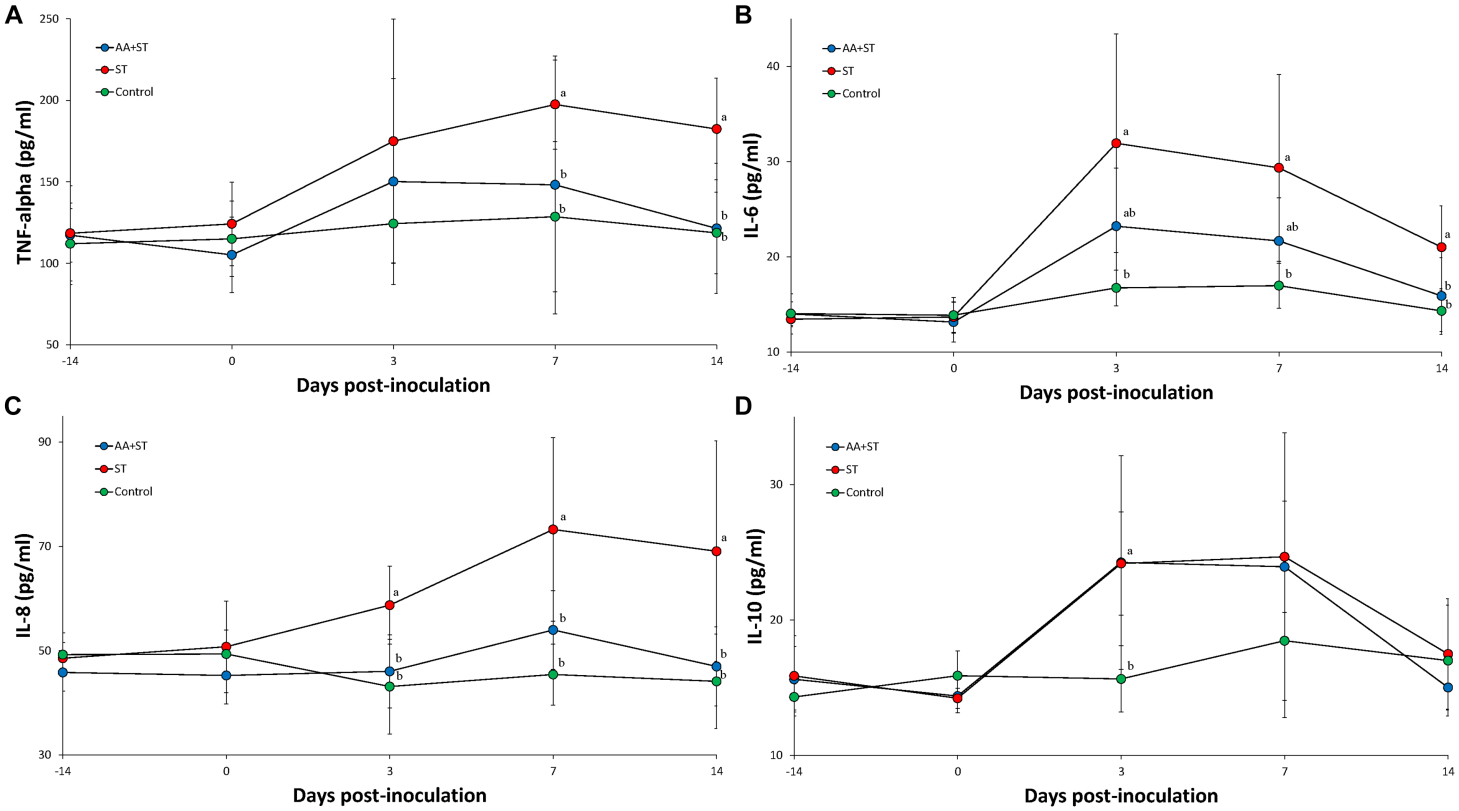
Levels of cytokines in serum. Variation is expressed as the standard deviation. **(A)** tumor necrosis factor-α. **(B)** interleukin-6. **(C)** interleukin-8. **(D)** interelukin-10. Pigs were fed a control standard diet supplemented with an amino acid blend, followed by oral inoculation with *Salmonella enterica* serovar Typhimurium (AA+ST group), oral inoculation with *Salmonella enterica* serovar Typhimurium without supplementation with an amino acid blend (ST group), and control pigs. Different letters within a sampling point mean statistically significant differences (*p* < 0.05).

Serum TNF-α levels were significantly (*p* < 0.05) lower in the AA+ST and control groups at 7 and 14 dpc than those from the ST groups (Figure 3A). Serum IL-6 levels were significantly (*p* < 0.05) higher in the ST group at 3, 7, and 14 dpc than those from the control group. Serum IL-6 levels were significantly (*p* < 0.05) lower in the AA+ST and control groups at 14 dpc than those from the ST group (Figure 3B). Serum IL-8 levels were significantly (*p* < 0.05) lower in the AA+ST and control groups at 3, 7, and 14 dpc than those from the ST group (Figure 3C). Serum IL-10 levels were significantly (*p* < 0.05) lower in the AA+ST and control groups at 3 dpc than those from the ST group (Figure 3D).

Superoxide dismutase activity.

**Figure 4:**
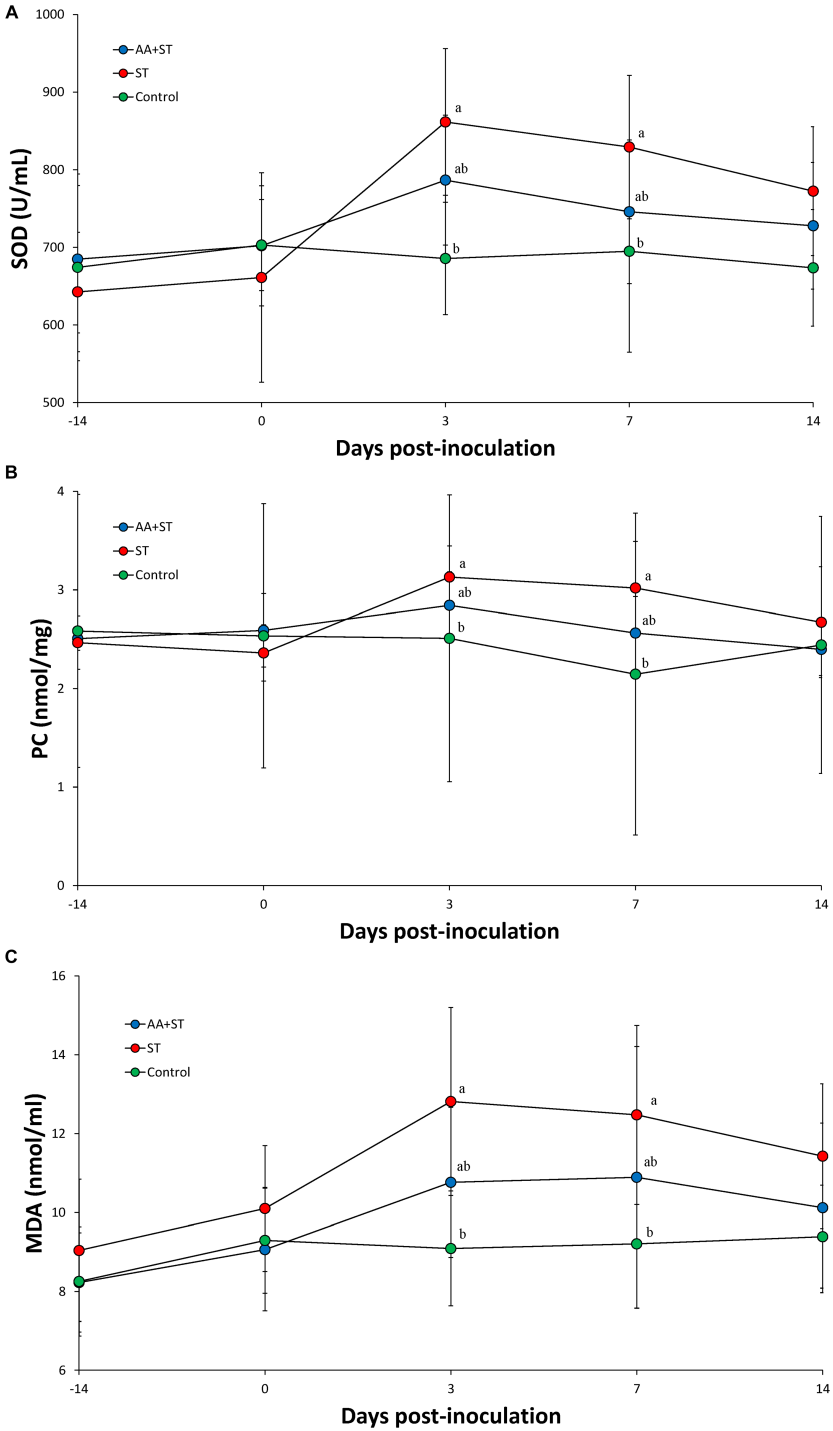
**(A)** Superoxide dismutase activity. **(B)** Levels of protein carbonyl in serum. **(C)** Levels of malondialdehyde in serum. Pigs were fed a control standard diet supplemented with an amino acid blend, followed by oral inoculation with *Salmonella enterica* serovar Typhimurium (AA+ST group), Pigs were oral inoculation with *Salmonella enterica* serovar Typhimurium without supplementation with an amino acid blend (ST group), and control pigs. Different letters within a sampling point mean statistically significant differences (*p* < 0.05).

Serum SOD activities were significantly (*p* < 0.05) higher in the ST group at 3 and 7 dpc than those from the control groups and tend to increase in the AA+ST and control groups at 3 and 7 dpc than those from the ST group (Figure 4A).

### Protein carbonyl and malondialdehyde assay

Serum protein carbonyl (Figure 4B) and malondialdehyde (Figure 4C) levels were significantly (*p* < 0.05) higher in the ST group at 3 and 7 dpc than those from the control groups and tend to increase in the AA+ST and control groups at 3 and 7 dpc than those from the ST group.

### Salmonella lesion score

**Figure 5:**
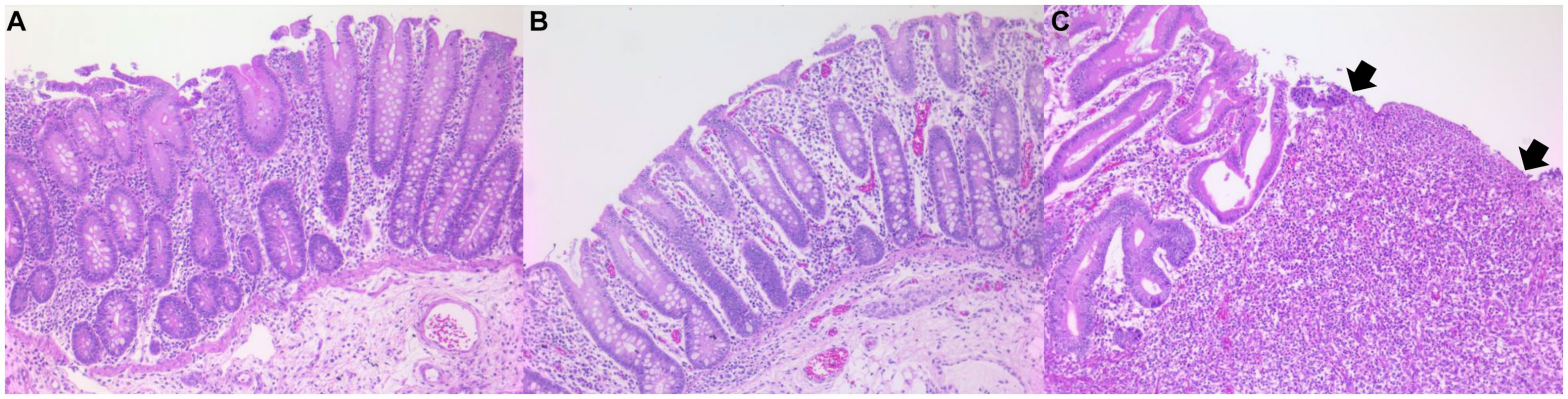
Histopathology in *Salmonella*-induced cecal lesions. **(A)** Mild cecal lesions with mild neutrophilic infiltrate in pigs were fed a control standard diet supplemented with an amino acid blend, followed by oral inoculation with *Salmonella enterica* serovar Typhimurium (AA+ST group). **(B)** Normal cecum in control pigs. **(C)** Severe cecal lesions with moderate neutrophilic infiltrate and ulceration (arrows) in pigs were oral inoculation with *Salmonella enterica* serovar Typhimurium without supplementation with an amino acid blend (ST group).

Pigs in the AA+ST (lesion score 1.28 ± 0.44, Figure 5A) and control lesion score 0 ± 0, Figure 5B) groups had significantly (*p* < 0.05) lower *Salmonella* lesion scores than that in the ST ((lesion score 2.05 ± 0.67, Figure 5C) group at 14 dpc. Pigs in the AA+ST group had significantly higher *Salmonella* lesion scores than those in the control group at 14 dpc.

## Discussion

Supplementation with an AA blend (Met + Thr + Trp) has been used successfully to improve gut health and immune function, thus potentially alleviating the negative effects of *Salmonella Typhimurium* infection on pigs. This experimental challenge study evaluated the growth performance of pigs through the measurement of ADWG. Due to the small number of pigs in each group and the shorter period of observation post-*S. typhimurium* challenge, a significant difference in ADWG between treated (AA + ST) and untreated (ST) groups was not observed.

Gastrointestinal disorders are identified through the biomarker measurement of inflammatory cytokines (22). Pigs that were experimentally infected with *S. typhimurium* exhibited a positive correlation of histological lesions with serum levels of TNF-α that were sensitive to tissue injury (23). The addition of an AA blend (Met + Thr + Trp) supplemented into pig feed increased the presence of an anti-inflammatory cytokine, IL-10, while reducing the amount of proinflammatory cytokines such as TNF-α, IL-6, and IL-8, as measured through serum samples from pigs experimentally challenged with *S. typhimurium*. The change in the amount of pro- and anti-inflammatory cytokines induced through feed supplementation with an AA blend reduced the severity of *Salmonella*-induced cecal lesions. Ample scientific evidence has been collected demonstrating that an AA blend used as an additive in pig feed is effective as an anti-inflammatory. Supplementation of methionine numerically increased the amount of anti-inflammatory IL-10 cytokine content in the jejunum of piglets experiencing intrauterine growth retardation (24). Of the three AAs used in supplementation, tryptophan blocks TNF-α-induced IL-8 secretion, which specifically provides strong anti-inflammatory effects on intestinal epithelial cells (25). The use of threonine supplementation in a high-fiber diet did not sufficiently maintain pig growth performance when challenged with *S. typhimurium*. (15). Supplementation of feed with an AA blend provided additional benefits, rendering it a great anti-inflammatory substance. It also reduced the severity of *Salmonella*-induced cecal lesions in pigs experimentally challenged with *S. typhimurium*. These results indicate that the supplementation of feed with an AA blend reduces intestinal inflammation and pathology in the pig.

*Salmonella* infection has been associated with the induction of oxidative stress, a condition characterized by an imbalance between the production of ROS and the cellular antioxidant defense mechanisms. This is manifested by an increase in oxidative stress markers such as MDA, indicating lipid peroxidation, and changes in antioxidant defenses, such as alterations in SOD activity. These molecular changes reflect the complex dynamics of the host–pathogen interaction and the impact of oxidative stress on cellular components. (2, 5).

To evaluate the anti-inflammatory activity of the AA blend, an *in vitro* examination was first performed using porcine intestinal epithelial cells. A cell-based *in vitro* evaluation system is a useful model to screen feed additive candidates for their functional effect and efficacy prior to an *in vivo* trial. In this study, the condition of intestinal inflammation was established by treatment with the combination of bacterial endotoxin (LPS) and mycotoxin (DON), or H_2_O_2_ to IPEC-J2s. *In vitro* studies demonstrated that pretreatment with the AA blend exerted a reduction of LPS/DON-induced inflammatory responses by downregulating IL-8 mRNA expression and ROS reduction under H_2_O_2_-induced oxidative stress. Furthermore, *in vivo* AA blend supplementation showed similar anti-inflammatory and anti-oxidative effects, especially reducing inflammatory cytokines, as expected in an *in vitro* study.

Salmonellosis is not currently well controlled by an effective strategy. Due to the large antigenic diversity of *Salmonella*, along with the fact that piglets are exposed extremely early in life (even in primary breeding herds), vaccination has produced mixed successes as a prevention and control strategy (26). With the emergence of antibiotic-resistant bacteria, antibiotics provide little efficacy against *Salmonella*. Treatment of salmonellosis is further hindered due to plasmids prevalent within *S. enterica* that encode for antimicrobial resistance (27). Various antibiotics have been used in therapeutic trials to treat severe *Salmonella* infections in pigs, but offer little merit (28). The Korean government began a restriction on antibiotic use as feed additives in 2005 due to overuse and misuse by producers. As a result, *Salmonella* control and management rely on alternative methodologies to traditional vaccination and antibiotic use. The results of this study demonstrate that the supplementation of feed with an AA blend was one of these successful alternative methods that could be used in the application of *S. typhimurium* control.

## Data availability statement

The original contributions presented in the study are included in the article/supplementary material, further inquiries can be directed to the corresponding author.

## Ethics statement

The animal study was approved by Seoul National University Institutional Animal Care and Use Committee. The study was conducted in accordance with the local legislation and institutional requirements.

## Author contributions

SH: Data curation, Formal analysis, Investigation. JS: Data curation, Formal analysis, Investigation. JK: Data curation, Formal analysis, Investigation. MG: Data curation, Formal analysis, Investigation. MP: Data curation, Formal analysis, Investigation. EO: Data curation, Formal analysis, Investigation. J-OM: Data curation, Formal analysis, Investigation. CC: Conceptualization, Methodology, Project administration, Supervision, Writing – original draft, Writing – review & editing.

## Footnotes

^1^ https://www.ncbi.nlm.nih.gov/tools/primer-blast/
